# A Case of Nonislet Cell Tumor Hypoglycemia Due to Metastatic Salivary Myoepithelial Carcinoma

**DOI:** 10.1016/j.aace.2024.06.002

**Published:** 2024-06-18

**Authors:** Margaret C. Slack, Samantha Sovich, Chana R. Sachs, Dorothy Martinez, Run Yu

**Affiliations:** 1 Department of Medicine, David Geffen School of Medicine at UCLA, Los Angeles, California; 2 Department of Pathology and Laboratory Medicine, David Geffen School of Medicine at UCLA, Los Angeles, California

**Keywords:** nonislet cell tumor hypoglycemia, insulin-like growth factor-2, salivary myoepithelial carcinoma, corticosteroids

## Abstract

**Background/Objective:**

Nonislet cell tumor hypoglycemia (NICTH) is an uncommon cause of hypoglycemia due to a relative surplus of insulin-like growth factor 2 (IGF-2) or its precursor molecule. The diagnosis is confirmed by an elevated ratio of IGF-2 to insulin-like growth factor 1 (IGF-1). Myoepithelial carcinoma (MECA) is a rare and aggressive salivary gland cancer that has not been previously associated with NICTH.

**Case Report:**

A 63-year-old female with a past medical history of metastatic salivary MECA, type 2 diabetes mellitus previously on metformin, hypertension, and hypothyroidism presented to her oncologist for chemotherapy and was found to have a serum glucose of 30 mg/dL (reference: 65-99). She was admitted for further diagnostic work-up which revealed an insulin level of <1 μU/mL (reference: 3-25), C-peptide <0.5 ng/mL (reference: 1.1-4.3), IGF-1 of 15 ng/mL (reference: 41-279), and IGF-2 of 147 ng/mL (reference: 180-580) with an IGF-2:IGF-1 molar ratio of 10, consistent with NICTH. The patient’s hypoglycemia unfortunately was quite resistant to treatment, requiring a combination of corticosteroids, continuous dextrose infusion, and somatostatin injections. The patient died 3 weeks after presenting with hypoglycemia.

**Discussion:**

Salivary MRCAs commonly contain pleomorphic adenoma gene 1 oncogene rearrangements which are associated with increased IGF-2 production and may predispose patients to hypoglycemia.

**Conclusion:**

This case demonstrates that NICTH can be associated with metastatic salivary MECA. The hypoglycemia in this scenario is challenging to manage and is associated with poor prognosis.

**Highlights**

• Salivary myoepithelial carcinoma (MECA) can cause nonislet cell hypoglycemia.
• An elevated IGF-2 to IGF-1 ratio greater than 10 supports a diagnosis of NICH.
• Corticosteroids, dextrose infusion, and somatostatin can be utilized to treat NICH.
• MECAs may contain fusion genes that predispose patients to NICH.

**Clinical Relevance**

This report describes the first published case of noninsulin mediated hypoglycemia (NICH) due to metastatic salivary myoepithelial carcinoma and discusses genetic changes in this particular malignancy that may predispose patients to the condition. We also review the treatment options for NICH and discuss possible adjunct therapies for refractory hypoglycemia.

## Introduction

Nonislet cell tumor hypoglycemia (NICTH) is a rare paraneoplastic phenomenon in which a tumor induces hypoglycemia through overproduction of insulin-like growth factor 2 (IGF-2) and its precursor molecule pro-IGF2. Severe hypoglycemia due to nonislet cell tumors have been reported in the literature since as early as 1930,^1^ though the etiology of hypoglycemia and pathophysiology of NICTH were not yet understood. Excess IGF-2 causes hypoglycemia by activating the insulin receptor-B isoform resulting in an insulin-like effect.^2^ Pro-IGF-2 or “big IGF-2” is also biologically active and significantly elevated in patients with NICTH.^3^ Pro-IGF-2 is thought to induce hypoglycemia by increasing the bioavailability of IGF-2 through altered binding with insulin-like growth factor–binding proteins and other associated proteins.^2^ Recurrent hypoglycemia in the setting of suppressed insulin, C-peptide, growth hormone, and beta-hydroxybutyrate levels is suggestive of NICTH.^2^ The diagnosis is further confirmed by an elevated molar ratio of IGF-2 to insulin-like growth factor 1 (IGF-1) greater than 10.^3^

NICTH is classically associated with mesenchymal and epithelial tumors, though has been associated with an increasing number of malignancies.^4^ The incidence and epidemiology is difficult to ascertain as the NICTH literature exists as individual case reports and small case series.^5^ Treatment of NICTH is surgical resection of the causative tumor when feasible and corticosteroids in nonoperable cases.^4^ Myoepithelial carcinoma (MECA) is a rare aggressive salivary gland cancer that has not been previously associated with NICTH. Genomic analysis shows that MECA tumors commonly contain pleomorphic adenoma gene 1 (*PLAG1*) rearrangements, which have been correlated with increased IGF-2 expression.^6^ Here we share the first reported case of NICTH due to MECA. We detail key findings that led to the diagnosis and discuss genetic changes in MECA tumors that may predispose patients to NICTH.

## Case Report

A 63-year-old female with a history of metastatic MECA originating in the left parotid gland, type 2 diabetes, hypertension, paroxysmal atrial fibrillation, and hypothyroidism presented to oncology clinic for chemotherapy infusion and was found to have a serum glucose of 30 mg/dL (reference: 65-99) on preinfusion labs. She was directed to the emergency room where she was stabilized with intravenous dextrose infusions. The patient had suffered several episodes of hypoglycemia at home over the past week in both the mornings and evenings, often after several hours of fasting. The episodes were characterized by confusion and tremulousness, confirmed with finger-stick glucoses in the 30s, and resolved with administration of sugar-containing foods, fulfilling Whipple’s triad. The patient’s only diabetes medication was metformin, which had been discontinued once the hypoglycemia began. Given concern for recurrent severe hypoglycemia, the patient was admitted to the hospital for evaluation and endocrinology was consulted.

Initial labs revealed normal renal function and stably elevated liver enzymes with normal bilirubin, international normalized ratio, and mildly decreased albumin. During an episode of hypoglycemia (serum glucose 33) the patient had a suppressed insulin level of <1 μU/mL (reference: 3-25), C-peptide <0.5 ng/mL (reference: 1.1-4.3), β-hydroxybutyrate level <1.0 (reference: <3), and random cortisol of 23 (reference: morning 8-25). Thyroid function testing revealed an elevated thyroid stimulating hormone of 8.3 (reference: 0.3-4.7) and a low-normal free T4 of 0.80 (reference: 0.80-1.70), and the patient’s levothyroxine dose was increased from 75 mcg to 88 mcg. IGF-1 was suppressed at 15 ng/mL (reference: 41-279) (1.96 nmol/L) and IGF-2 was also decreased at 147 ng/mL (reference: 180-580) (19.6 nmol/L). Despite the low IGF-2 level, there was high suspicion for NICTH given the IGF-2: IGF-1 molar ratio of 10.

**Fig. 1:**
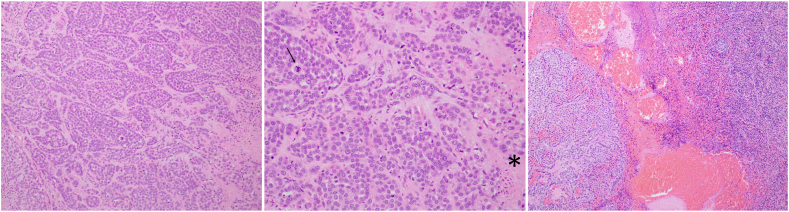
Histologic sections of high-grade myoepithelial carcinoma (hematoxylin and eosin stain). *Left*, Invasive nests and cords of malignant myoepithelial cells with prominent eosinophilic basal lamina (100×). *Middle*, High-grade features are prominent, including nuclear pleomorphism, increased mitotic activity, atypical mitoses (arrow), and necrosis (∗) (200×). *Right*, Metastatic myoepithelial carcinoma to a neck lymph node (100×).

**Fig. 2:**
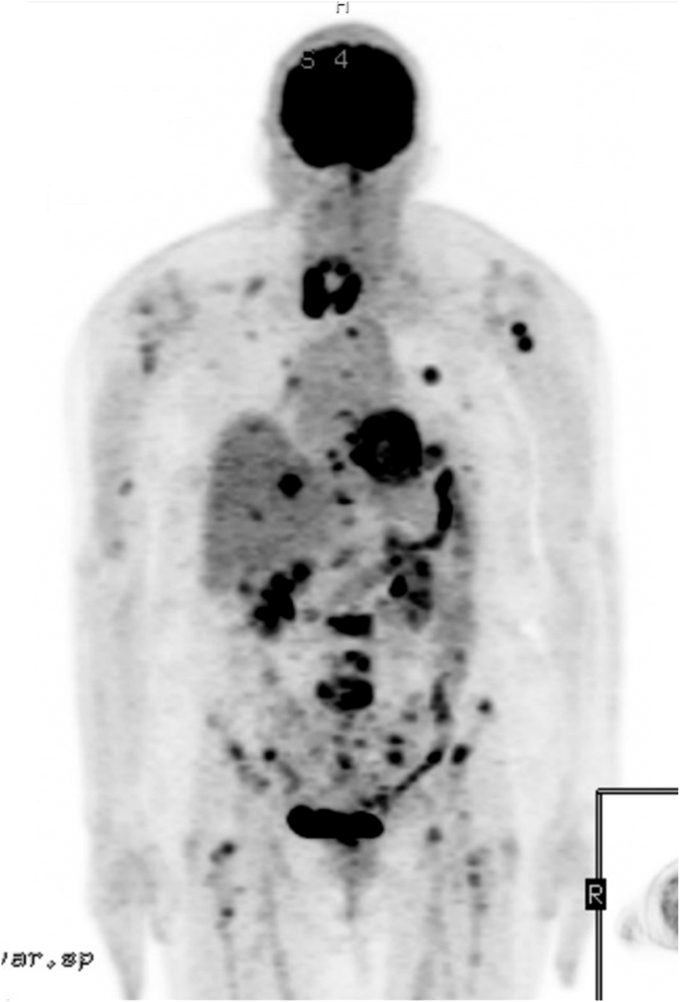
Maximal intensity projection image of fludeoxyglucose positron emission tomography. Note the primary salivary myoepithelial carcinoma in the neck and extensive visceral and bony metastases.

The patient had been diagnosed with salivary cancer approximately 1 year prior after noticing a rapidly enlarging left facial mass. An initial positron emission tomography scan showed an fludeoxyglucose-avid parotid mass with associated lymphadenopathy and no distal metastasis. Surgical excision with lymph node dissection confirmed a diagnosis of stage 4A high-grade MECA involving 2 of 26 dissected neck lymph nodes (Fig. 1). The patient received 30 fractions of adjuvant radiation. A repeat positron emission tomography scan 8 months after diagnosis revealed new diffuse disease including bilateral pulmonary nodules, innumerable osseous lesions, and liver lesions (Fig. 2). Chemotherapy with carboplatin and paclitaxel was initiated and the patient was presenting for cycle 6 when she was sent to the emergency room for hypoglycemia.

Given the patient’s diffusely metastatic disease unamenable to resection, the patient was started on steroid therapy with dexamethasone, which was ultimately titrated up to 5 mg twice daily due to persistent hypoglycemia. Repeat labs obtained after steroid initiation showed an increase in IGF-1 to 27 and a stable IGF-2 level of 144 suggesting appropriate response to therapy. Despite steroid therapy, the patient required continuous infusion of dextrose to maintain euglycemia. To prevent volume overload, the infusion was concentrated to dextrose 20% with the rate increased to 60 ccs per hour. Due to continued hypoglycemia, somatostatin was initiated as adjunct therapy based on a case report of a patient with NICTH who was effectively treated with a combination of somatostatin and corticosteroids.^7^ After a 12-day hospitalization, the patient elected to transition to home hospice. Dexamethasone and dextrose 20% were continued outpatient, but somatostatin was not prescribed due to insurance authorization delays. She died 8 days after discharge.

## Discussion

This case represents the first reported incidence of NICTH due to MECA and details a clinical course complicated by recurrent hypoglycemia highly resistant to treatment.

NICTH should be considered in the differential diagnosis of hypoglycemia in patients with known malignancy after medication-induced hypoglycemia and insulinoma have been excluded. The patient’s low insulin, C-peptide, and β-hydroxybutyrate levels during an episode of hypoglycemia indicated a noninsulin mediated process. The diagnostic considerations for this patient included impaired gluconeogenesis in the setting of metastatic liver involvement, excess glucose utilization by tumor, and NICTH. Impaired gluconeogenesis was considered as the patient had multiple bulky liver metastases and a significant transaminitis; however. this was felt to be unlikely as the patient’s liver synthetic function was intact. Ultimately, the patient’s clinical course and elevated IGF-2:IGF-1 ratio provided further clarity.

During the hospitalization, the patient experienced recurrent hypoglycemia despite treatment with steroids, continuous dextrose infusion, and somatostatin. This refractory hypoglycemia bolstered the diagnosis of NICTH, as hypoglycemia due to impaired hepatic gluconeogenesis or excess glucose consumption by tumor would be expected to resolve with continuous dextrose infusion. Furthermore, the patient’s IGF-2:IGF-1 ratio of 10 was highly suggestive of NICTH. A normal IGF-2:IGF-1 molar ratio is 3:1.^5^ patients with NICTH often have ratios of 10:1 or greater.^5^ While pro-IGF2 levels are not commercially available,^5^ if obtained, an elevated pro-IGF2 level would have further supported the diagnosis. A glucagon stimulation test was also not conducted but may be of use when there is diagnostic uncertainty, as a markedly inadequate response raises the likelihood of impaired gluconeogenesis due to liver dysfunction.^8^

Treatment of NICTH consists of surgical excision of the tumor whenever possible, as NICTH is cured by complete tumor resection.^2^ When resection is not feasible, debulking and embolization of large tumors should be considered to treat hypoglycemia, even in cases where it does not impact overall survival.^4^ In cases of inoperable NICTH, corticosteroids are recommended and efficacious in reducing hypoglycemia.^9^ Corticosteroids are hypothesized to reduce hypoglycemia by decreasing circulating levels of pro-IGF2.^9^ A small observational study by Teale et al^10^ confirmed a measurable reduction in pro-IGF2 levels in patients with NICTH treated with corticosteroids. While high doses of dexamethasone did reduce this patient’s insulin-like growth factor ratio, she continued to experience hypoglycemia. Somatostatin has been proposed as an adjunct therapy and trialed with inconsistent results. Several studies have shown failure of somatostatin analogs to prevent hypoglycemia, possibly due to lack of functional somatostatin receptors on NICTH-inducing tumors.^2^ However, there are other reports of patients who benefited from combination therapy with corticosteroids and somatostatin analogs and required lower maintenance steroid doses.^7^ Given the lack of data, it is reasonable to consider somatostatin as an adjunct treatment in cases of NICTH refractory to steroid monotherapy. It is unclear what benefit somatostatin provided this patient as she received only 3 doses.

NICTH has been associated with a growing number of malignancies, potentially due to increasing recognition of the condition. MECA is a rare aggressive malignancy that is diagnosed in <1% of salivary tumors^11^ and has not been previously associated with NICTH. Genomic analysis of MECA tumors has revealed high rates of oncogenic fusion genes with frequent involvement of *PLAG1*,^6^ which encodes a zinc finger transcription factor involved in cell proliferation.^12^ Tumors with *PLAG1* rearrangements demonstrate increased production of IGF-2, and it is thought that this is one of the main methods by which PLAG1 exerts its oncogenic effects.^6^ Dysregulation of *IGF-2* expression occurs in many other cancers, and IGF-2 is thought to stimulate cancer development via binding to IGF-1 receptors and subsequent activation of the MAPK and PI3-K/Akt pathways.^6^ In addition to tumorigenesis, IGF-2 and its precursor molecules have the potential to induce hypoglycemia leading to NICTH. Further research is needed to determine if patients with MECA may be at particularly high risk of NICTH because of these mutations.

This case demonstrates that MECA may induce NICTH. The hypoglycemia in this scenario is challenging to manage and is associated with poor prognosis.

## Disclosure

The authors have no conflicts of interest to disclose.
